# Perceived causes of severe mental disturbance and preferred interventions by the Borana semi-nomadic population in southern Ethiopia: a qualitative study

**DOI:** 10.1186/1471-244X-12-79

**Published:** 2012-07-12

**Authors:** Solomon Teferra, Teshome Shibre

**Affiliations:** 1 Department of Psychiatry, College of Health Sciences, Addis Ababa University, Addis Ababa, Ethiopia; 2 Division of Psychiatry, Umeå University, Umeå, Sweden

## Abstract

**Background:**

Culture affects the way people conceptualize causes of severe mental disturbance which may lead to a variation in the preferred intervention methods. There is a seemingly dichotomous belief regarding what causes severe mental disturbance: people living in western countries tend to focus mainly on biological and psychosocial risk factors; whereas, in non-western countries the focus is mainly on supernatural and religious factors. These belief systems about causation potentially dictate the type of intervention preferred. Studying such belief systems in any society is expected to help in planning and implementation of appropriate mental health services.

**Methods:**

A qualitative study was conducted among the Borana semi-nomadic population in southern Ethiopia to explore perceived causes of severe mental disturbance and preferred interventions. We selected, using purposive sampling, key informants from three villages and conducted a total of six focus group discussions: three for males and three for females.

**Results:**

The views expressed regarding the causes of mental disturbance were heterogeneous encompassing supernatural causes such as possession by evil spirits, curse, bewitchment, ‘exposure to wind’ and subsequent attack by evil spirit in postnatal women and biopsychosocial causes such as infections (malaria), loss, ‘thinking too much’, and alcohol and khat abuse. The preferred interventions for severe mental disturbance included mainly indigenous approaches, such as consulting Borana wise men or indigenous healers, prayer, holy water treatment and seeking modern mental health care as a last resort.

**Conclusions:**

These findings will be of value for health care planners who wish to expand modern mental health care to this population, indicating the need to increase awareness about the causes of severe mental disturbance and their interventions and collaborate with influential people and indigenous healers to increase acceptability of modern mental health care. It also provides information for further research in the area of mental health in this semi-nomadic population.

## Background

Severe mental illnesses are universal phenomena in the world affecting every society, but beliefs about causation vary across cultures. It has been reported that people living in western countries focus mainly on biological and social risk factors such as genetic vulnerability, disease of the brain, infection or stressful social conditions or personal weakness [1-4], but the predominant views held by people living in non-western countries focus mainly on supernatural and religious factors [5-9]. In a cross-cultural study comparing Australia and Japan, infection, allergies and genetics were the most commonly attributed causes of mental illness reported in Australia; whereas, nervousness and perceived constitutional weakness were more often reported in Japan [3]. Another comparative study of young adults in Hong Kong and England found that, while the Hong Kong youths believed that social factors were the likely cause of schizophrenia, the English youths were more likely to report genetic factors as a cause [1]. Similarly, in reports from Germany and Italy, lay people held a predominantly biological view of the cause of schizophrenia [2,4]. A report from Turkey showed about 60% of a rural population held the view that personal weakness might be a cause of schizophrenia [10]. In contrast to this, in Bali, Indonesia, the majority of families held the belief that schizophrenia was caused by supernatural causes, such as witchcraft or disturbance by spirits [11]. Similarly, a study exploring the belief system surrounding causes of symptoms of mental illness in a primary care setting in Saudi Arabia reported that patients attributed their symptoms to religious and supernatural factors, saying they could be the result of punishment from Allah [12]. Despite these seemingly dichotomous views regarding attribution, a significant proportion of people living in western countries still endorse the spiritual and magical views. For instance, a study done in Italy by Magliano et al reported that 4% of the participants, including lay people, professionals, and relatives, believed magic, spirit possession and spells as causes of schizophrenia [2].

Beliefs about the causes of mental illnesses influence the preferred treatments [13,14]. People living in non-western countries mainly rely on indigenous healing practices which are defined as ‘helping beliefs and strategies that originate within a culture or society and that are designed for treating the members of a given cultural group, [15]. A report from Nigeria showed the majority of people preferred indigenous over the modern health care system which was consistent with their predominant belief about causes of mental illnesses [16]. A key informant study from Butajira, rural Ethiopia, also showed a preference for indigenous practices for mental disorders and modern medicine is more preferred for somatic conditions [7]. In contrast to this, in the USA, beliefs in biological causes (i.e., chemical imbalance, genes) are associated with endorsement of professional, biologically focused treatments (e.g., prescription medication, psychiatrists, and mental hospital admissions) [17].

Although several studies have investigated beliefs about causes of mental illnesses in a variety of non-western countries, including some African countries, studies involving nomadic populations are extremely rare. A community based epidemiological study done among Borana semi-nomadic people in southern Ethiopia near the Kenyan boarder, involving 1854 people of both sexes aged 15 years and above, using Composite International Diagnostic Interview (CIDI) failed to show any case of psychosis [18]. But, using a qualitative method, the Borana people recognized and reported cases of severe mental illness which were later confirmed to have psychosis by diagnostic interview [19]. In this study, we explored the perceived causes of severe mental disturbance and the preferred intervention methods when a member of their community developed severe mental disturbance. As the Ministry of Health in Ethiopia plans to expand mental health care services, by integrating it into the primary health care delivery system, we hope this study will generate useful information for health care providers as well as policy makers. It was also hoped to generate useful hypothesis for further research.

## Methods

### Setting

The Borana semi-nomadic people reside in Southern Ethiopia close to the Kenyan Border, Borana administrative zone of Oromia regional state which is the largest regional states in Ethiopia. A significant number of the Borana people also live in Northen Kenya who share the same language and culture. They are among the few nomadic people that still exist in the world. The zone covers a vast predominantly arid and savannah vegetation of 48, 743 sq kms. The population of Borana zone is 966,467, consisting of 489,001 males and 477,466 females, with about 9% of the population classified as urban [20], of this 77% are Borana Oromos and remaining other ethnic groups. They follow predominantly indigenous religious beliefs although Protestant religion has attracted more followers recently [21]. The indigenous Oromo belief involves a monotheistic belief system which refers to a single creator god *Waaqa*. They believe also in spirit coming from this god which can dwell in humans, animals and objects. They have a system of indigenous healing practices involving different rituals. The Borana have their own indigenous governance system, known as the *Gada* system. It is a model African-born democratic governance system where there is peaceful transition of power every eight years to elected leaders, albeit a male dominated system. Family is highly valued in Borana, and children and women are highly protected. Although the practice is being discouraged because of the risk of HIV infection, polygamy is common, and women can have extramarital affair as well, without the husband being annoyed.

Gender roles exist in Borana society with women mainly engaged in household activities, looking after smaller animals, milking, fetching water and collecting fire wood, while the men engage in managing cattle and farmland [22].

The infrastructure is poorly developed, and they have little access to modern mental health care but access to primary health care has improved recently.

The Borana pastoralists move from place to place in search of grazing land and water for their cattle**,** especially during severe periodic drought seasons, which occur as frequently as every two to three years. This study was conducted in the villages of Megado, Dida Yabello and Dida Hara and, according to data from register of local administrations the total population aged over 18 years in the three districts was 10,598. These villages were the site of an earlier epidemiological study [18].

### Study design and selection of participants

In total, 56 Key Informants (KI) were invited to participate in a qualitative study, each selected using purposive sampling, the most common sampling method in qualitative research [23]. Six Focus Group Discussions (FGDs) each composed of between eight and 10 participants were organized across the three study sites or villages. The inclusion criteria for the FGD participants/KIs were: men and women of age 18 years and above, resident in the village for over two years, and formal or informal community leaders, traditional leaders, *Aba Ollas* (head of village) or generally respected individuals with whom the researcher could communicate for information exchange (e.g. Community Health Agents). Visits to the study sites were made prior to the date of the FGDs to ensure that all the villages were represented in the groups.

Based on the inclusion criteria, two FGDs, one for men and one for women, were organized in each study site.

### Interview procedure

The FGDs were conducted by a moderator and attended by a note-taker, both of whom are Ethiopian psychiatrists who speak the local Borana dialect (Oromiffa language). The group discussions were held in a local heath care center (two FGDs) or community meeting area (four FGDs). The interview followed a topic guide using a series of open-ended questions regarding awareness of mental illness, symptoms and causes of severe mental illnesses, and traditional ways of caring for severely mentally ill persons in the context and culture of the Borana pastoralists. Each FGD lasted between 45 and 80 minutes. In all groups, the moderator encouraged participants to become actively involved in the discussion and ensured that each participant had an equal chance to contribute. An audio tape was used for recording the meeting and supplemented by hand-written notes.

### Data analysis

A person fluent in the local dialect was hired to transcribe the audio recordings and notes from the FGDs. The local Oromiffa transcripts were then translated into English by the first author (ST), who is a psychiatrist, and the accuracy of the translations was cross-checked by the coauthor (TS), a psychiatrist who has good command of both languages.

### Codes, themes and categories

Our analysis method was ‘thematic/content analysis’ a process which involves systematically examining the data and other notes in order to identify themes and develop categories [24]. We went through the process of systematically reducing the massive raw data to identify concepts and themes relating to our research question i.e. identifying how the participants conceptualize severe mental disturbance in relation to causes and what kinds of interventions they prefer for the severely mentally ill.

The two authors independently coded each transcription one of them using the Open Code software [25] (ST) and the other (TS) manually based on the preference of the author. Multiple coding was considered to be one of the methods to maintain rigor in qualitative research [24]. The coding was predominantly ‘close to the text’ using the participants own descriptions. The codes were then grouped into categories. Any discrepancies were discussed and consensus on the appropriate coding reached. Anonymous quotes were used to illustrate the facts.

### Ethical considerations

Ethical clearance was obtained from the Faculty of Medicine Research and Publication Committee, Addis Ababa University, and the Ethiopian Science and Technology Agency. Participants were fully informed about the purpose of the study, and all of them gave their verbal consent to participate in the study.

## Results

Themes referring to causes of severe mental disturbance such as bewitchment, spirit possession and curse were grouped in one core category ‘supernatural’ and other themes such as infections, substance abuse and loss were grouped under a core category ‘biological and psychosocial’. Themes referring to preferred intervention methods were grouped into three big core categories namely ‘indigenous’, ‘religious’ and ‘modern mental health care’. These themes and categories helped us to provide an overall picture of each interview. As such there was no striking difference reported between the male and female participants. So, we didn’t present the opinions of each separately, only an indication of sex of the participant is given besides the quote. The findings of the study are presented in two parts: (1) conceptualization and perceived causes of mental disturbance among the Borana semi-nomadic community, and (2) preferred interventions when someone from their community develops mental illness.

### Conceptualization and perceived causes of severe mental disturbance

When participants were asked what name was given to someone who had severe mental disturbance, they unanimously agreed on one name, *marata* [the *Borana Oromo* word for ‘mad’ which is slightly derogatory]. Several reasons were given for a person to become *marata*. Traditional beliefs dominated the expressed conceptualizations of mental illness. But, interestingly, there were several biological and psychosocial factors mentioned as causing *marata*. It is worth noting here that most participants didn’t have a firm opinion regarding a single cause. Changes in position were noted as the participants engaged in heated discussion. Often several factors were mentioned as important in causing *marata*. These causes can be grouped in to two broad categories:

### Supernatural influences

#### Bewitchment

The majority of participants spoke of the importance of supernatural influences in causing severe mental disturbance. Bewitchment, witchcraft and possession by evil spirits were all reported to be causes of mental disturbance. The concept of bewitchment was particularly related to the evil deeds of others, in order to retaliate to an offence or misdeed. According to the participants, when someone is bewitched, it not only makes the person develop severe mental disturbance, it also causes property loss. And the mental illness could also be transmitted to other people related with the person who is affected. Severe mental disturbance was believed to be caused by god as reported by one of the participants ‘Madness is first caused by *Waqa* [god].’[**FGD 3;** R8, female]. Madness was also believed to be caused by a curse from the Borana spiritual leaders as illustrated by one male participant:

FGD2; R1 (male): In Borana there is such thing called *murma* [translated as bewitchment]. When one has *murma* [bewitchment], it is difficult to tell who did it. It can happen to him even when he walks on the street. When this thing befalls him, it may make him *marata* [the Oromo word for ‘mad’]…. It may happen to him when he takes it from someone else. The Borana spiritual leaders may curse someone and this may make him mad. If someone takes [steals] someone else’s money or material, the person who lost the money or material may do something in retaliation which makes the other person mad. He may do some witchcraft on him which may make him mad. People would say he became mad because he took someone else's property.

#### Evil spirit attack

The following situations were particularly mentioned as making people more vulnerable for attack by evil spirits:

#### Child birth

Many participants reported that ‘exposure to wind’ before a woman becomes clean from the blood after child birth to be a reason for attack by evil spirits. This idea was reflected particularly by female participants for obvious reasons. Some were reported to have recurrent episodes with each child birth experience as illustrated by the following quote.

"FGD 6; R5 (female): We have seen many kinds of madness. There is a woman who became mad after giving birth to a child. In Borana, such kind of madness is believed to be caused by exposure to wind before the woman becomes clean [vulnerable to evil spirit attack]. My own sister had developed such kind of madness. I took her to many indigenous healers. She is well now. She is completely free. She gave birth to other children afterwards. We are born from the same mother."

#### Exposure to blood, war and dirty water

Another cause of mental disturbance that was reported by the participants was exposure to blood. The experience of fear when seeing blood was reported to be the reason for developing the disturbance. War was also mentioned as a cause of mental disturbance. Fear from crossing flooding river [during rainy season] was also mentioned to be a cause for mental disturbance. Although stress might be an important factor here, these incidents are believed to involve some bad spiritual interference as well. So, it combines both psychosocial stressor and spiritual influence. The following quotes illustrate the above points:

"FGD1; R2 (male): When he has nose bleeds, the wind will take the blood. The blood will be changed to headache. Because of this, his head will be disturbed. The other reason is fall from accident. Even after falling from a car, the blood may be carried away by the wind. [FGD1] R3: [interrupting R2] and when someone stands by the side of blood in the sun."

"FGD 3; R5 (female): …Madness comes from two causes: when they see this dirty water. The girl who was mad and difficult to handle became mad when she tried to cross the kobo river [a river found in the area], which was full. She saw the volume of the water and became afraid. This girl would become better when taken to health facility, but her illness comes back. Her problem was caused by that river. When this mad girl is given some tablets, her condition improves, but she becomes ill again. Now she is totally mad. Sometimes she becomes better. That is how it comes."

#### Biological and psychosocial factors

##### Infections, loss: ‘worry’ vs. ‘true madness’, ‘curable vs. non-curable’

Many participants across the group meetings mentioned malaria (a tropical infectious disease) as an important cause of mental disturbance, but they distinguished mental disturbance caused by malaria, which they considered to be a curable condition, from other forms of worry and madness, generally considered a severe and non-curable condition. Another important distinction emerging from the discussions was between so-called ‘true madness’ and ‘worry’. According to their description, ‘worry’ was considered to be more related to psychosocial stressors as a result of loss, such as death of loved ones, loss of property and such events.

The following quote illustrates this belief:

"FGD2; R6 (female): Mental illness has two types. The mental illness caused by malaria is an illness and it improves with medicine. There is another type of mental illness which is the true ‘marata’ [Borana name for ‘mad’]. Such person is truly mad and his mind is disturbed [lit “his mind is turned inside out”]. There is another variety which is worry. … This is different from madness. Worry and madness are two different things. Worry comes from loss of property in the family. … In madness, the mind becomes totally changed. Worry refers to thinking too much in Borana. Worry comes from ‘thinking too much’. If you had wealth and lose that wealth, worry comes. But the truly mad throws off his clothes and walks naked. It could be man or woman, people take a different path away from him."

#### Alcohol and khat

Alcohol and khat, a naturally occurring amphetamine-like substance whose leaf is chewed to get the stimulant effect, were considered to play a role in causing mental disturbance by some of the participants.

"FGD 6; R7 (male): Yes. These things [khat and alcohol] are like poison [NB: R7 himself was chewing khat while saying this]. God gives rain one day, and at other times it becomes dry. Just like that people may not always get khat or alcohol all the time. If they get money to buy these things, they may become mad."

"FGD6; R5 (male): As far as this alcohol is concerned, the person who drinks this thing is already mad. There is some guy who wouldn’t hesitate to kill his mother when he is intoxicated with alcohol. He had to leave his mother alone, but he wouldn’t do so. This is madness caused by alcohol. Drinking alcohol is madness, more than chewing khat."

#### Madness may be inherited

Some of the participants reported that madness could be inherited from parents to children. But compared with the other reasons, this one was mentioned only by few participants. One of the participants gave the following example to illustrate the heritability of mental disturbances:

"FGD 3; R3 (female): The child of a mad person may or may not be mad. There is an old lady known by the name … She was married to a man who wanted to have children. She gave birth to two children. They become mad whenever they drink alcohol. Their madness is obvious. She [their mother] is mad. She escapes to the jungle and then comes back to her home. She sometimes talks to people; at other times she ignores people."

#### Preferred interventions for mental disturbance

The participants in the various FGDs were asked about preferred interventions among the Borana pastoralist community when someone develops mental illness. The findings were summarized as follows. Several interventions methods were described by the participants. Although they expressed preferences for one intervention over another, their approach was pragmatic, pursuing every possible means of treatment until they got solutions. The intervention methods included indigenous, religious, and modern mental health care. They are summarized under three categories as follows:

#### Borana wise men and indigenous healers

The overwhelming majority of the participants said they would take their relatives to Borana wise men for consultation. These wise men were recognized by the participants as having the necessary skill to help people who developed mental disturbance arising from a range of problems. The following quote illustrates some of the techniques they use in helping such patients:

"**FGD 1** R5 (male): There are some people who dislike their own homes. Medical treatment does not help for such problem. Such people are better taken to Borana wise men and they reveal what is buried in their houses. They give instructions that it should be removed from the house. For such people they give a medicine and instruct the person to wash her body with it [referring a woman in his neighborhood who had undergone the ritual]. It usually helps. He also requests the person to come back to him. He repeats that same order. She was freed and lives peacefully like you and me."

The second most preferred intervention for the mentally ill was indigenous healers. Such healers use a variety of different methods to treat people who have mental disturbance. This was described as follows:

"FGD 6; R3 (male): [When someone becomes mad] The first thing that is done in Borana is to perform a ritual to exorcise the spirit. They believe the person's madness is caused by zar [possession by spirit that is believed to have ancestral origin]. The person may not show improvement. They will not take him to a health facility. They will say that the person has murma [bewitchment] and will consult an indigenous healer, taking coffee and tobacco with them [as payment for his service]."

#### Prayer, Holy water: ‘The devil will scream and leave the patient!’

Seeking help from religious institutions which offer prayer and treatment with holy water were strategies reported by several participants. It was reported that consulting religious institutions, such as Christian and Islam prayer places is common in this predominantly animist population signifying the more pragmatic approaches they follow as illustrated below.

"FGD3; R3 (female): We will take him to prayer place. If he is very sick, it may be possession by the devil. He might scream. If he refuses the prayer and asks for traditional ritual, we might take him to the place where they exorcise the spirit by ritual. If these interventions fail, I will take him to the indigenous healer, and he might give him some herb to wash his body with or to rub his body with or fumigate with. I will do these things even if he refuses… If all the above means fail, he will be taken to hospital. If all fail to help him become well, he will be left to be on his own. He will move around and may live in the jungle like a beast, just like we said earlier."

"FGD 6; R4 (male): It is like what has been said. Some people try hard with kalichas [Muslim wise men]. These kalichas would say the person was attacked by Satan that came out of termite hills [common in the area] and would do different things. Some people may be freed from the illness after they removed Satan from them."

#### Modern health care as a last resort

Modern health care was mentioned by a few participants who said it was usually a last resort when other modes of interventions had failed. Again the majority had a more pragmatic approach ready to try anything that would help the sick person.

"FGD 3; R3 (female): There will also be a traditional ritual ceremony to intercede with the spirit. They make coffee, and perform rituals [dancing]. Yet others will take him to an indigenous Borana healer to deal with the evil deeds of others [witchcraft]. When all this fails they will take him to hospital. They will take him to Addis Ababa [the capital of Ethiopia where they find the only specialized mental hospital in the country]. They will try all these means and if he fails to improve, they will leave him alone [depending on his condition the patient has two choices: either to remain with his family where he would be provided with his basic needs or leave his village and wander in the streets of towns being homeless]."

## Discussion

This qualitative study explored views held by prominent men and women from the Borana semi-nomadic community in Southern Ethiopia with regards to their belief about causes of severe mental disturbances and their preferred intervention modalities when someone in their community developed mental disturbance. It provides a unique opportunity to understand the belief systems and intervention in mental health related issues in this relatively isolated and underserved community.

According to Kleinman [26], culture is an important factor affecting how people perceive severe mental disturbance. There is a general belief that people from western countries and non-western countries have different views regarding the causes of mental disturbances, the former being more biologically-oriented with the latter tending to emphasize religious-magical views. Studies done in western countries about public views regarding causes of mental illnesses reported the predominantly held beliefs to be biological, such as genetics or infections, and social factors such as stressful life events, traumatic experiences, family problems, and social disadvantage[1-4].

The predominant views held in this study population regarding causes of mental disturbance incorporated both religious/magical and biopsychosocial. Among the religious/magical views, possession by evil spirits was commonly held as the main cause of mental disturbance. Bewitchment/witchcraft was another magical view that was reported as a common reason for developing mental disturbance. But besides the religious/magical views, there were prominent biopsychosocial causes reported: biological causes such as malaria, traumatic brain injury, epilepsy, and alcohol and khat abuse; psychosocial stressors such as loss (of property or family member), the experience of child birth, severe psychological stress (such as fear, war) and ‘thinking too much’ were reported time and again by the participants as major causes of mental disturbance. ‘Thinking too much’, which was repeatedly mentioned across the groups as cause of mental disturbance, is a finding consistent with reports from Zimbabwe by Abas et al [27] and Uganda by Okello & Ekblad [28] which found a similar attribution style referring to less severe forms of mental distress such as the non psychotic conditions referred to as ‘worry’.

Mental disturbances following the experience of child birth which was associated with ‘exposure to wind before the woman becomes clean’ was a common view held by the participants which is consistent with a finding from a study in a rural setting in Butajira, Ethiopia. The women participants reported pregnancy to be a time of high risk for evil spirit attack. To protect the woman from attack by evil spirit, they keep the woman in hiding behind curtain and close every hole or window until she becomes clean [29]. Mental illnesses were reported to be heritable by a small number of the participants. Although we put it under biological, it was not very clear from their description whether heritable meant inheriting through genes or spirits. It is good to bear in mind that the Borana people believe in the influence of ancestral spirits descending on subsequent generations.

Several reports from other non-western countries also showed the diversity of opinion held by people living in non-western countries. For instance, a study done in Nigeria involving a large community survey found that as many as one third of the respondents suggested that possession by evil spirits could be a cause of mental illness, but in this same study the majority held the biopsychosocial causes such as drug and alcohol misuse, traumatic event/shock, stress, physical abuse and genetic inheritance as the causes of mental illness [30]. Another study done among adults in a rural community in 250 adults residing in Karfi village in northern Nigeria reported that one third of the respondents reported drugs of abuse as causes of major mental illness and another third reported divine wrath or spirit possession as the cause[16]. In a cross-sectional survey done in Pakistan involving 404 people at the outpatient departments of Aga Khan University Hospital Karachi, reported that more than half of the respondents held spiritual or magical causes for psychosis, less than half of the participants reported loneliness or unemployment as a cause for psychotic symptoms [31].

More recent studies from Ethiopia showed inclusion of biological and psychosocial factors as causes of mental disturbances in addition to the age old spiritual and magical views. For instance, a study done in western Ethiopia before the 1974 communist revolution on traditional perception and treatment of mental disorders reported that traditional and religious views were the predominant views as causes of mental illnesses. These were disturbances in relationships between people and divinity, possession by evil spirits or punishment by God to the unfaithful [32]. But a recent survey in a nearby area, a small town in western Ethiopia, reported biopsychosocial problems such as poverty, stress and drug abuse were believed to be important problems for mental illness besides religious/magical views such as God’s will or attack by evil spirit [6,8]. Mulatu also reported a similar finding of predominantly psychosocial and supernatural retribution as causes of mental than physical illnesses in North-western Ethiopia challenging the earlier report that lay Ethiopians exclusively believe in spiritual factors as causes of mental illnesses [6,33].

The findings in this study regarding preferred interventions for mental illness were mainly indigenous, although modern health care was also mentioned as important. The overwhelming majority reported preference for consulting the Borana wise men and indigenous healers, but they also reported their belief in other interventions such as modern mental health care delivered in health centers in their area or going to bigger towns to get more specialized care as a last resort. According to their report the care delivered in modern health facility was expensive and inaccessible. To go to a hospital one has to sell his property and take the patient to the capital city, Addis Ababa, which is very far from the place they live. This shows that the people do not necessarily have a special preference of one over the other; rather, it all depends on the availability of the services, financial capacity and severity of the problem as demonstrated by the pragmatic nature of their approach to seeking help, although the role of belief system in dictating preference is acknowledged. According to reports from Nigeria and India, belief system had a direct effect on the preferred treatment. In these countries, the prevailing attribution styles were reported to be mainly supernatural and the preferred treatments were mainly alternative i.e. traditional and religious [34,35]. But other reports showed a significant majority of people preferred modern health care despite their attribution styles. For instance, in the study done in rural Northern Nigeria, nearly half of the respondents preferred orthodox medical care for the mentally sick while a third were more inclined to spiritual healing [16]. In the study done in Pakistan, again nearly half of the respondents reported psychiatric consultation to be the single most important management step [31]. This shows people living in non-western countries endorse modern western medical care for mental health problems in addition to the existing indigenous methods. They tended to be more pragmatic and pluralistic in their approach and were will willing to try anything that would help the sick. Their views tend to be more dynamic and amenable to change as the situation demands which is similar to other findings from non-western countries [13].

## Strengths and limitations of the study

The strength of this study is the fact that it presents an important knowledge on the views of the understudied Borana semi-nomadic population in Ethiopia regarding their explanatory systems of causes of mental disturbances with special emphasis to religious/existential magical frameworks and interactions of the different systems and their preferred interventions. Qualitative studies offer a unique opportunity to describe the lived experiences of people and it is an important tool to investigate mental health related phenomena. The findings in this study have the potential to serve as hypotheses to investigate other similar populations living in underserved remote areas. It will also be useful for policy makers to design appropriate mental health intervention strategies. The main limitation of this study is generalizability. However, this is the inherent nature of a qualitative study design since the study participants are selected by the investigators ‘purposefully’. Hence the views expressed are those of the participants and may not necessarily reflect the views held by every member of the community in the area. But, to get as many diverse views as possible, we selected at least one participant from each village and made sure that even all men and women participants were from a different village. So, there may not be significantly different views left untold. Another potential source of bias is the researchers’ educational background of being psychiatrists. Maximum possible care was taken to focus mainly on describing the thoughts of the participants and distance ourselves from introducing our opinion when presenting the results.

## Conclusion

In sum, we found that even people who live in the remote parts of southern Ethiopia had mixed views regarding the causes of severe mental disturbances. They reported various biological, psychological and social factors as causes for severe mental disturbance besides the religious, spiritual and magical factors. They also reported the role of modern health care in helping people with severe mental disturbances. But, their preferred intervention was mainly indigenous healers, religious rituals and other non-medical interventions because these were the modality of treatments they had easy access to in their locality. Interestingly, they were highly pragmatic in their attitude and willing to try every possible means until the patient got relief. Modern mental health care was mentioned as a last resort. An important explanation for this could be lack of access to modern mental health care which is almost non-existent in the area. The argument that people in African do not utilize modern mental health care services may be a gross underestimation of the people’s ability to identify what is best for them. This view may further weaken the already neglected field of mental health care by governments. Indigenous and alternative treatments will continue to be important for helping people with mental health problems, but this shouldn’t preclude the expansion of mental health service to communities in these countries. It is hoped that the findings in this study will give the critical areas to address while attempting to introduce and expand modern mental health care in this community. Designing a mental health policy and strategy that incorporates culture is important [36]. The role of traditional (indigenous) medicine is appreciated in Ethiopia’s health policy and stated as ‘Due attention shall be given to the development of the beneficial aspects of Traditional Medicine including related research and its gradual integration into Modern Medicine’ [37]. This needs to be extended to indigenous mental health interventions as well. Educating the public and close working collaboration with traditional institutions will be necessary when planning expansion of mental health service to the community which is likely to be endorsed.

## Competing interests

The authors declare that they have no competing interests.

## Authors’ contributions

TS participated in the conception and design of the study. TS secured the grant to conduct the study. ST and TS participated in data collection and analysis. TS and ST contributed in the analysis and write up of the manuscript. Both authors were involved in the write up of the final manuscript.

## Pre-publication history

The pre-publication history for this paper can be accessed here:

http://www.biomedcentral.com/1471-244X/12/79/prepub
